# PocketOptimizer 2.0: A modular framework for computer‐aided ligand‐binding design

**DOI:** 10.1002/pro.4516

**Published:** 2023-01-01

**Authors:** Jakob Noske, Josef Paul Kynast, Dominik Lemm, Steffen Schmidt, Birte Höcker

**Affiliations:** ^1^ Department of Biochemistry University of Bayreuth Bayreuth Germany; ^2^ Computational Biochemistry University of Bayreuth Bayreuth Germany; ^3^ Present address: Department of Physics University of Vienna Vienna Austria

## Abstract

The ability to design customized proteins to perform specific tasks is of great interest. We are particularly interested in the design of sensitive and specific small molecule ligand‐binding proteins for biotechnological or biomedical applications. Computational methods can narrow down the immense combinatorial space to find the best solution and thus provide starting points for experimental procedures. However, success rates strongly depend on accurate modeling and energetic evaluation. Not only intra‐ but also intermolecular interactions have to be considered. To address this problem, we developed PocketOptimizer, a modular computational protein design pipeline, that predicts mutations in the binding pockets of proteins to increase affinity for a specific ligand. Its modularity enables users to compare different combinations of force fields, rotamer libraries, and scoring functions. Here, we present a much‐improved version––PocketOptimizer 2.0. We implemented a cleaner user interface, an extended architecture with more supported tools, such as force fields and scoring functions, a backbone‐dependent rotamer library, as well as different improvements in the underlying algorithms. Version 2.0 was tested against a benchmark of design cases and assessed in comparison to the first version. Our results show how newly implemented features such as the new rotamer library can lead to improved prediction accuracy. Therefore, we believe that PocketOptimizer 2.0, with its many new and improved functionalities, provides a robust and versatile environment for the design of small molecule‐binding pockets in proteins. It is widely applicable and extendible due to its modular framework. PocketOptimizer 2.0 can be downloaded at https://github.com/Hoecker-Lab/pocketoptimizer.

## 1 INTRODUCTION

Ligand binding is essential in most biological processes, for example, enzyme catalysis, immune recognition, regulation of metabolism, cellular signal transduction, or control of gene expression. The ability to design such interactions will help us address many of society's current challenges. Computational tools for the design of small molecule‐binding pockets in proteins are of great interest for the design of tailored enzymes that can catalyze reactions for which no natural catalyst exists^1^, ^2^, ^3^, ^4^ or for the development of specific biosensors that can detect small molecules in vitro and in vivo.^5^, ^6^

From an energetic point of view, the recognition of small molecules by proteins relies on the cooperative formation of a set of weak, non‐bonded interactions, primarily van der Waals (vdW), and electrostatic attraction, as well as the formation of hydrogen bonds. These interactions can be estimated based on a variety of receptor ligand scoring functions^7^, ^8^, ^9^ and can be used to identify specific mutations that lead to increased binding affinity of a protein to its ligand. Additionally, solvent effects have been discussed to play a major role but are not always included in the scoring functions.^10^, ^11^, ^12^ Apart from protein–ligand interactions, internal protein interactions must also be considered upon mutation to minimize destabilizing effects on the protein structure. To this end, we developed a modular pipeline called PocketOptimizer that accounts for both packing energies and binding‐related energies and that can include different scoring functions to allow adaptation to specific design problems.^13^ In this design pipeline, we address side chain flexibility via rotamer libraries and ligand flexibility by using stochastic or systematic search algorithms.^13^, ^14^ In addition, discrepancies between designs and experimental results can be more easily determined because all sampled conformations, together with the computed interaction energies, are written to user‐inspectable files. Finally, a deterministic solving procedure is applied to extract the optimum from the sampled search space.^15^

Due to the significance of protein–ligand binding, several tools have been developed to computationally score and (re)design protein binders. Commonly, these techniques attempt to approximate binding free energy changes and binding constants based on ensembles of bound and unbound states.^16^, ^17^, ^18^, ^19^ While most programs use only one way of designing and scoring, the PocketOptimizer framework, which only evaluates the bound state, is set up to use different modules. This way, different approaches or scoring functions can be compared, and a tailored method can be created for the design problem at hand. However, PocketOptimizer became outdated, making the addition of new functions difficult. Here, we present a new version, PocketOptimizer 2.0. Its new user interface is much more accessible, and the modular architecture has been improved and extended to provide more options for modeling and scoring within the computer‐aided design process.

## 2 RESULTS

### 2.1 Design pipeline

**FIGURE 1:**
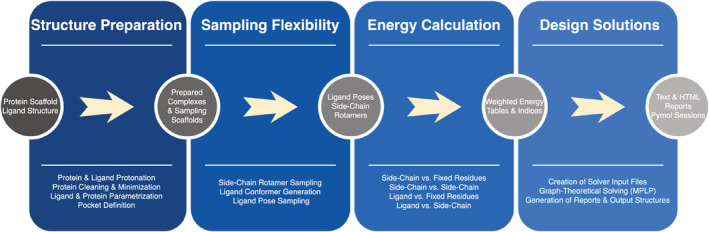
The different steps of the PocketOptimizer pipeline. For each section, the required input, the included steps, and the obtained output are listed. The workflow starts with a protein and a ligand structure. These are processed in a preparation step (first box). To account for flexibility, rotamers and ligand poses are sampled (second box). Next, the interaction energies for each rotamer and each ligand pose against the fixed scaffold and against each other are calculated (third box). Finally, the best design solutions are identified using an integer linear programming solving algorithm (fourth box)

The design pipeline can be divided into four main steps: structure preparation, flexibility sampling, energy calculations, and computation of design solutions (see Figure 1). As input for the pipeline, the structures of a protein and a ligand are needed. The ligand has to be placed manually inside the binding pocket since its initial position influences the design results and can therefore hardly be automated. Before the actual design process can start, the protein undergoes a cleaning procedure to remove unwanted ions, water molecules, small molecules, and protein chains. Next, all amino acid side chains are protonated according to a pH value defined by the user. Afterwards, an initial minimization step is performed to resolve potential clashes that may occur in the process of model building. During minimization, backbone atoms are typically constrained to maintain the backbone conformation. Once scaffold preparation is complete, the binding pocket can be defined by selecting flexible residues at certain design positions. Thus, all non‐selected residues are fixed along with the backbone. Similar to the protein, the ligand is protonated. This is then followed by a parameterization step in which atom types, force field parameters, and partial charges are assigned for both structures.

In the second step, the flexibility sampling step of the pipeline (Figure 1), rotamers for residues at all defined design positions and ligand poses can be sampled. PocketOptimizer 2.0 includes two rotamer libraries: a smaller, backbone‐independent rotamer library compiled from high‐resolution protein crystal structures named CMLib^20^ and a larger, backbone‐dependent rotamer library known as the Dunbrack rotamer library.^21^ For ligand pose sampling, ligand conformations can be generated using different algorithms.^13^, ^14^ All generated conformers are then systematically translated and rotated along a user‐defined grid to create an ensemble of poses within the binding pocket. To reduce computational overhead, rotamers and poses are subsequently pruned from the search tree.

Interaction energies for rotamers and ligand poses are calculated in the third step of the pipeline. For this purpose, the binding pocket needs to be decomposed into self‐ and pairwise interaction energies. Whereas self‐interaction energies describe the interaction between either rotamers or ligand poses and the fixed scaffold, pairwise‐interaction energies describe the interaction between rotamers or ligand poses and other rotamers. This decomposition of energies allows solving the design problem at a later stage. The computed energies can be further subdivided into those representing interactions within the protein or interactions between protein and ligand. While the so‐called packing‐related energies represent changes in protein stability, the binding‐related energies represent changes in binding affinity and are therefore particularly important. Hence, they can be scaled according to the packing energies and calculated based on a variety of receptor‐ligand scoring functions.^8^, ^9^, ^22^

In the last step of the pipeline, PocketOptimizer uses a solver algorithm based on integer linear programming (ILP) to identify the best design solutions.^15^ The algorithm requires weighted energy tables and indices of all rotamers and ligand poses. Once executed, it can provide indices that minimize the total energy. The corresponding rotamers and ligand poses represent the global minimum energy conformation (GMEC) of our system, where the ligand binding energy can again be extracted. According to these rotamers and ligand poses, output files of the resulting energies and design structures can be generated. This includes the designed structures in PyMOL sessions^23^ as well as the text and HTML files containing the generated energy tables.

### 2.2 New features in PocketOptimizer 2.0

The original version of PocketOptimizer 1.0^20^ was mainly a collection of binaries and Python scripts that interconnected the various parts of the design pipeline. It was then extended with a command‐line interface to allow for easier interaction with the framework.^24^ Nonetheless, source code and software dependencies remained unchanged. As, these are now a decade out of date, we fundamentally rewrote the software and implemented a range of new functionalities to extend it further (Table 1). This resulted in version 2.0 of PocketOptimizer, which will be presented in a comparative manner in the following section.

**TABLE 1:** Comparison of PocketOptimizer 1.0 and 2.0, listing the main differences between both versions

	Version 1.0	Version 2.0
Language	Python 2.7/C++	Python 3.9
UI	CLI	API/CLI
Processing	Single core	Multi core
Scaffold preparation	External (Chimera)	Internal (*systemPrepare*)
Ligand preparation	External	Internal (OpenBabel/antechamber/MATCH)
Minimization	External	Internal (OpenMM)
Rotamer sampling	TINKER	*FFEvaluate*
Rotamer library	CMLib	CMLib/Dunbrack
Energy computation	BALL	*FFEvaluate*
Force field	AMBER96	AMBER ff14SB/CHARMM36
Scoring options	CADDSuite/Vina	Smina/*FFEvaluate*
Compute detection	Re‐computation	Detection of computed elements
Time estimation	None	Progress bars

Abbreviations: API, application programming interface; CLI, command‐line interface.

The first version of PocketOptimizer was written in Python 2.7, which lost maintenance support in the beginning of 2020. Since most Python libraries are no longer supporting Python 2.7, PocketOptimizer was rewritten in Python 3.9. Additionally, we implemented a Python application programming interface that allows not only to use specific functionalities of the design pipeline, but also permits a more user‐friendly and flexible interaction with the framework. PocketOptimizer 2.0 now also offers multi‐core processing, making it faster and scaling better on a larger number of CPUs. Moreover, progress bars have been added to monitor computation progress. Additionally, parts that have already been computed can be now reused when varying a design task.

Previously, in PocketOptimizer 1.0, the user had to prepare the input protein structures, often using external software such as Chimera.^25^ In the new version of our software, HTMD's protein preparation pipeline *systemPrepare* has been implemented^26^ for this. It also comes with the possibility to assign specific protonation states according to calculated empirical pKa values (PROPKA^27^) and user‐defined pH values. After preparation, minimization is now also available using the molecular dynamics framework OpenMM,^28^ which provides GPU‐accelerated minimization. In addition, we implemented a small molecule preparation interface that uses the OpenBabel chemical toolbox^29^ for protonation and the Antechamber software,^30^ or MATCH^31^ for parameterization.

Rotamer sampling previously relied on the molecular modeling software TINKER.^32^ During the procedure, clashing rotamers were minimized to induce a better fit. Since this minimization can distort the resulting rotamers and is based on an older force field version, we replaced TINKER with the force field evaluation tool *FFEvaluate*.^33^ For the same reason and to further limit external dependencies, the Biochemical Algorithms Library (BALL),^34^ previously used for all energy calculations, was replaced by *FFEvaluate*. Whereas for BALL, all atom types had to be manually predefined for the AMBER96 force field, *FFEvaluate* handles them through a Python library called ParmEd,^35^ allowing the usage of newer force fields such as AMBER ff14SB or CHARMM36. In addition, the scoring function for ligand interactions has been adapted. While version 1.0 included CADDSuite^36^ and AutoDock Vina,^8^ CADDSuite has been removed due to its dependency on the BALL library. AutoDock Vina, on the other hand, is now included in Smina,^22^ which is a new fork and includes other scoring functions such as Vinardo (Vina RaDii Optimized).^9^ These scoring functions differ in their compilation of scoring terms describing effects such as vdW interactions, electrostatics, and solvation. Besides, *FFEvaluate* has been implemented for binding‐related energy calculations based on force fields that are also used to evaluate internal protein interactions. Accordingly, the enhancements and improvements not only make the pipeline more consistent, but also make it less reliant on the use of external software.

### 2.3 Benchmarking

PocketOptimizer 1.0 was validated against a benchmark compiled from the 2010 version of the PDBbind database.^37^, ^38^ Complexes were selected based on the availability of a high‐quality crystal structure with no mutations outside of the binding pocket, only minor conformational differences of the backbone in the binding pocket, with less than seven potential binding water molecules, and with less than 15 rotatable bonds in the ligand. According to these selection criteria, a benchmark set consisting of 12 differently folded proteins had been compiled.^20^ For each protein, at least two mutational variants with a corresponding affinity measure for the same ligand were included. To validate the new version of PocketOptimizer, we compiled a subset based on this original benchmark. Pairs of mutational variants with at least a 50‐fold difference in binding affinity were selected. This difference in binding affinity was considered to be well outside of experimental error and should be predicted by our design pipeline. In addition, we extended the benchmark set with new structures from the 2020 version of the PDBbind database using the same selection criteria. Overall, our new benchmark set consists of 13 different proteins and 33 protein crystal structures (see Table S1). Skeletal representations of all ligands included in the compiled benchmark set are shown in Figure S1.

### 2.4 Backbone‐dependent rotamers lead to improved prediction accuracy

We tested PocketOptimizer 2.0 against the extended benchmark set to compare both versions of the software. The results indicate a similar performance, with a mean prediction accuracy of about 66% compared to about 71% in the first version (see Table 2). Significant differences were found only in two test cases, namely, neuroamidase N1, where the new version gave significantly better predictions, and purine nucleoside phosphorylase, where it made significantly worse predictions. In both test cases, this has been attributed to the fact that minimizing rotamers with TINKER led to a general preference for larger amino acids, as they can engage in more favorable interactions. We can largely overcome this bias by using a backbone‐dependent rotamer library and performing no subsequent rotamer minimization, which leads to a prediction accuracy of 70% (see Table 2). Looking only at the cases tested with both versions, PocketOptimizer 2.0 with the new rotamer sampling method achieves a higher overall prediction accuracy of 75% according to the total energies. If only the binding‐related energies are considered, this trend becomes even clearer, with the original rotamer library and sampling procedure achieving a prediction accuracy of about 71%, while the new sampling method and library lead to a higher prediction accuracy of about 84%. This is particularly evident in the case of purine nucleoside phosphorylase, where the original rotamer sampling procedure and library were only able to correctly predict one out of four cases, whereas our new rotamer sampling procedure in combination with the Dunbrack rotamer library leads to correct predictions in all cases. eroid isomerase, LAOBP: lys In this test case, the relevant design position is at the entrance of the binding pocket and assumed to influence binding dynamics, as it also has a high temperature factor.^39^ Three different variants were tested with PocketOptimizer 2.0: Histidine, aspartate, and phenylalanine, with the histidine showing significantly higher binding affinity. Like the first, the new version correctly predicts hydrogen bonds between ligand and aspartate. For histidine, this is the case only when we use our new sampling procedure and backbone‐dependent rotamers. Nonetheless, phenylalanine is rotated toward the ligand, regardless of rotamer sampling, and forms favorable vdW interactions, whereas it points away from the ligand in the crystal structure (see Figure S2).

**TABLE 2:** Correctly ranked design mutation pairs

Test case	Original sampling procedure and library (PocketOptimizer 2.0)		New sampling procedure and library (PocketOptimizer 2.0)		Original data (PocketOptimizer 1.0)	
	Total	Binding	Total	Binding	Total	Binding
D7r4 amine‐binding protein	1/1	1/1	1/1	1/1	1/1	1/1
ABC transporter alpha‐glycoside‐binding protein	0/2	0/2	1/2	1/2	−/−	−/−
Estrogen receptor α	1/1	1/1	1/1	1/1	1/1	1/1
FimH Fimbrial adhesin	2/2	2/2	2/2	2/2	−/−	−/−
HIV‐1 protease	5/5	5/5	4/5	5/5	5/5	5/5
Ketosteroid isomerase	2/2	1/2	2/2	2/2	2/2	2/2
Lysine‐, arginine‐, ornithine‐binding periplasmic protein	7/10	9/10	7/10	9/10	−/−	−/−
Neuroamidase N1	3/4	2/4	2/4	2/4	1/4	0/4
Nopaline‐binding periplasmic protein	1/2	1/2	0/2	1/2	−/−	−/−
Purine nucleoside phosphorylase (PNP)	1/4	0/4	4/4	4/4	7/8	6/8
Streptavidin	5/5	4/5	5/5	5/5	5/5	5/5
Thymidylate synthase (TS)	0/4	3/4	1/4	2/4	1/6	0/6
Anionic trypsin 2	1/2	2/2	1/2	2/2	1/2	1/2
Mean	65.9%	70.5%	70.5%	84.1%	70.6%	61.8%

*Note*: This is shown for two different versions of PocketOptimizer 2.0 using two rotamer sampling procedures in combination with two different rotamer libraries and for the original data from benchmarking with PocketOptimizer 1.0 (Vina). For PNP and TS, the number of pairs differs since we were more stringent in applying the cutoff of a 50‐fold affinity change for each pair. For each test case, the total number of design mutation pairs and the number of correctly ranked pairs by total energy or by binding energy are indicated. The mean value refers to the number of correct predictions in relation to the total number of predictions made.

**FIGURE 2:**
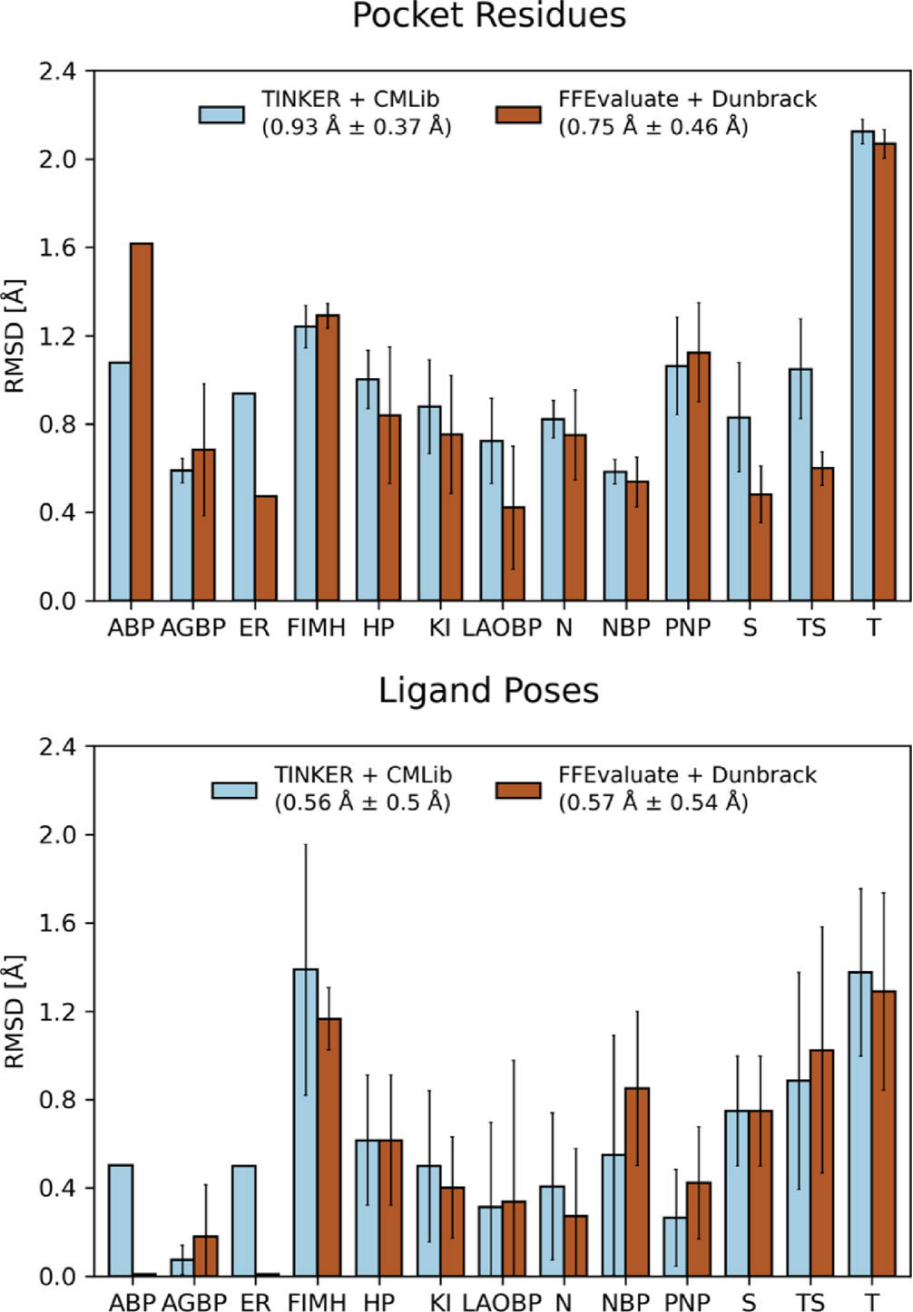
Pocket residue and ligand pose RMSD values between experimentally determined and designed structures. RMSD values were calculated after superimposing the structures using their backbone atoms. Only heavy atoms were considered in all calculations, and only residues that were allowed to change conformations during the designs were included. For each protein test case that included more than one crystal structure, the average RMSD and standard deviation were calculated. Protein test cases are ABP: D7r4 amine‐binding protein, AGBP: ABC transporter alpha‐glycoside‐binding protein, ER: estrogen receptor α, FIMH: fimH fimbrial adhesin, HP: HIV‐1 protease, KI: ketosteroid isomerase, LAOBP: lysine‐, arginine‐, ornithine‐binding periplasmic protein, N: neuroamidase N1, NBP: nopaline‐binding periplasmic protein, PNP: purine nucleoside phosphorylase, S: streptavidin, TS: thymidylate synthase, T: anionic trypsin 2

To gain further insight, we calculated structural deviations between experimentally determined and designed structures. On the one hand, we focused on the designed pocket residues, and on the other, on the predicted ligand poses (see Figure 2). We found that the pocket side chains deviate by 0.93 Å on average when designed with the original rotamer sampling method and library, while they deviate by only 0.75 Å with our new method and library. Not only the affinity predictions are more accurate overall, but also the pocket side chains are better reproduced on average. The ligand poses, on the other hand, are more comparable, differing by 0.56 Å on average with the original procedure and by 0.57 Å when *FFEvaluate* and Dunbrack are used. Nevertheless, significantly better pose predictions are observed for two test cases (ABP and ER). This indicates an overall good prediction of poses by PocketOptimizer. However, since the ligand starting poses were taken from the initial structures (see calculations) and often differ only slightly between mutants, the structural deviations may be higher than the suggested values.

## 3 CONCLUSION

PocketOptimizer 2.0 has been updated and refined to predict affinity‐improving mutations and to design protein–small molecule interactions. Different functions, such as scoring, can be easily compared, and approaches can be optimized for a specific design task. The program provides a clean user interface. Its compute times have been significantly improved by adapting the pipeline to multicore processing. The preparation of the protein scaffold and the ligand are now included in the pipeline, as well as a minimization step. To extend the modularity of the pipeline, we added the options for rotamer libraries, scoring functions, and force fields. In addition, rotamer sampling and energy calculations have been updated with newer tools. This improved version of PocketOptimizer performs as good or even better than its predecessor on an extended benchmark set. Overall, the affinity predictions appear to be more accurate, and also the pocket side chains are better reproduced on average. Thus, PocketOptimizer 2.0 provides a robust and versatile framework for the design of small molecule‐binding pockets in proteins.

## 4 MATERIALS AND METHODS

Protein and ligand structures were taken from the PDB, and ligand starting poses were assumed to be the same as in the crystal structures. Protonation states were adjusted according to the pH values reported in the literature for affinity measurements (see Table S1). Side chains were minimized with the AMBER ff14SB force field and allowed to change conformations during designs if they were within 4 Å of the ligand or a Cα atom of a mutation position. Residues located at the end of protein segments or involved in disulfide bridges were kept static. The number of ligand conformations was selected according to the number of rotatable bonds a ligand contains. Ligand poses were then created by rotating all generated conformations by ±20° around each axis and translating them by ±0.5 Å in each direction. Rotamer sampling was performed using two different procedures. First, TINKER in combination with the CMLib rotamer library and the AMBER96 force field was used, and second, *FFEvaluate* in combination with the Dunbrack rotamer library, and the AMBER ff14SB force field was used. Of the rotamers and ligand poses generated, only those with a vdW energy of less than 100 kcal/mol in the scaffold were kept. Protein–protein interactions were assessed based on the AMBER ff14SB force field, while protein–ligand interactions were evaluated using the Autodock Vina scoring function and were upscaled by a factor of 50. According to the objective of the design, predictions were considered to be correct if, after the identification of the GMEC, the binding energy of the mutant that experimentally shows higher binding affinity is lower.

## AUTHOR CONTRIBUTIONS

**Jakob Noske:** Formal analysis (equal); methodology (equal); validation (equal); writing – original draft (equal); writing – review and editing (equal). **Josef Paul Kynast:** Methodology (equal); validation (equal); writing – review and editing (equal). **Dominik Lemm:** Methodology (equal); validation (equal). **Steffen Schmidt:** Formal analysis (equal); methodology (equal); validation (equal); writing – review and editing (equal). **Birte Höcker:** Conceptualization (equal); formal analysis (equal); funding acquisition (equal); writing – original draft (equal); writing – review and editing (equal).

## CONFLICT OF INTEREST

The authors declare no potential conflict of interest.

## Supporting information


**Appendix S1:** Supporting Information

## Data Availability

PocketOptimizer 2.0 is available under the GNU general public license v3.0 under the following URL: https://github.com/Hoecker-Lab/pocketoptimizer.

## References

[1] Jiang L , Althoff EA , Clemente FR , et al. De novo computational design of retro‐aldol enzymes. Science. 2008;319(5868):1387–1391. 18323453 10.1126/science.1152692 PMC3431203

[2] Röthlisberger D , Khersonsky O , Wollacott AM , et al. Kemp elimination catalysts by computational enzyme design. Nature. 2008;453(7192):190–195. 18354394 10.1038/nature06879

[3] Siegel JB , Zanghellini A , Lovick HM , et al. Computational design of an enzyme catalyst for a stereoselective bimolecular Diels‐Alder reaction. Science. 2010;329(5989):309–313. 20647463 10.1126/science.1190239 PMC3241958

[4] Liu DS , Nivón LG , Richter F , et al. Computational design of a red fluorophore ligase for site‐specific protein labeling in living cells. Proc Natl Acad Sci U S A. 2014;111(43):E4551–E4559. 25313043 10.1073/pnas.1404736111 PMC4217414

[5] De Los Santos ELC , Meyerowitz JT , Mayo SL , Murray RM . Engineering transcriptional regulator effector specificity using computational design and in vitro rapid prototyping: Developing a vanillin sensor. ACS Synthetic Biology. 2016;5(4):287–295. 26262913 10.1021/acssynbio.5b00090

[6] Herud‐Sikimić O , Stiel AC , Kolb M , et al. A biosensor for the direct visualization of auxin. Nature. 2021;592(7856):768–772. 33828298 10.1038/s41586-021-03425-2 PMC8081663

[7] Morris GM , Huey R , Lindstrom W , et al. AutoDock4 and AutoDockTools4: Automated docking with selective receptor flexibility. J Comput Chem. 2009;30(16):2785–2791. 19399780 10.1002/jcc.21256 PMC2760638

[8] Trott O , Olson AJ . AutoDock Vina: Improving the speed and accuracy of docking with a new scoring function, efficient optimization, and multithreading. J Comput Chem. 2010;31(2):455–461. 19499576 10.1002/jcc.21334 PMC3041641

[9] Quiroga R , Villarreal MA . Vinardo: A scoring function based on autodock vina improves scoring, docking, and virtual screening. PLoS One. 2016;11(5):e0155183. 27171006 10.1371/journal.pone.0155183 PMC4865195

[10] Katkova EV , Onufriev AV , Aguilar B , Sulimov VB . Accuracy comparison of several common implicit solvent models and their implementations in the context of protein‐ligand binding. J Mol Graph Model. 2017;72:70–80. 28064081 10.1016/j.jmgm.2016.12.011 PMC5313374

[11] Gopal SM , Klumpers F , Herrmann C , Schäfer LV . Solvent effects on ligand binding to a serine protease. Phys Chem Chem Phys. 2017;19(17):10753–10766. 28116375 10.1039/c6cp07899k

[12] Nguyen NT , Nguyen TH , Pham TNH , et al. Autodock Vina adopts more accurate binding poses but Autodock4 forms better binding affinity. J Chem Inf Model. 2020;60(1):204–211. 31887035 10.1021/acs.jcim.9b00778

[13] Obabel . Generate multiple conformers—Open Babel 3.0.1 documentation. [cited 2021 Jul 2]. Available from: https://open-babel.readthedocs.io/en/latest/3DStructureGen/multipleconformers.html

[14] O'Boyle NM , Vandermeersch T , Flynn CJ , Maguire AR , Hutchison GR . Confab––Systematic generation of diverse low‐energy conformers. J Chem. 2011;3(1):8. 10.1186/1758-2946-3-8 PMC3073927 21410983

[15] Sontag D , Meltzer T , Globerson A , Jaakkola T , Weiss Y . Tightening LP relaxations for MAP using message passing. Proceedings of the 24th Conference on Uncertainty in Artificial Intelligence, UAI 2008. 2008 p. 503–510.

[16] Traoré S , Allouche D , André I , et al. A new framework for computational protein design through cost function network optimization. Bioinformatics. 2013;29(17):2129–2136. 23842814 10.1093/bioinformatics/btt374

[17] Barlow KA , Ó Conchúir S , Thompson S , et al. Flex ddG: Rosetta ensemble‐based estimation of changes in protein–protein binding affinity upon mutation. J Phys Chem B. 2018;122(21):5389–5399. 29401388 10.1021/acs.jpcb.7b11367 PMC5980710

[18] Hallen MA , Martin JW , Ojewole A , et al. OSPREY 3.0: Open‐source protein redesign for you, with powerful new features. J Comput Chem. 2018;39(30):2494–2507. 30368845 10.1002/jcc.25522 PMC6391056

[19] Panel N , Villa F , Opuu V , Mignon D , Simonson T . Computational design of PDZ‐peptide binding. Methods in Molecular Biology. 2021;2256:237–255. 34014526 10.1007/978-1-0716-1166-1_14

[20] Malisi C , Schumann M , Toussaint NC , Kageyama J , Kohlbacher O , Höcker B . Binding pocket optimization by computational protein design. PLoS One. 2012;7(12):e52505. 23300688 10.1371/journal.pone.0052505 PMC3531388

[21] Shapovalov MV , Dunbrack RL . A smoothed backbone‐dependent rotamer library for proteins derived from adaptive kernel density estimates and regressions. Structure. 2011;19(6):844–858. 21645855 10.1016/j.str.2011.03.019 PMC3118414

[22] Koes DR , Baumgartner MP , Camacho CJ . Lessons learned in empirical scoring with smina from the CSAR 2011 benchmarking exercise. J Chem Inf Model. 2013;53(8):1893–1904. 23379370 10.1021/ci300604z PMC3726561

[23] Schroedinger . The PyMOL Molecular Graphics System, Version 1.2r3pre, Schrödinger, LLC.

[24] Stiel AC , Nellen M , Höcker B . PocketOptimizer and the design of ligand binding sites. Methods in Molecular Biology. 1414. Humana Press Inc.; 2016. 63–75. 27094286 10.1007/978-1-4939-3569-7_5

[25] Pettersen EF , Goddard TD , Huang CC , et al. UCSF chimera––A visualization system for exploratory research and analysis. J Comput Chem. 2004;25(13):1605–1612. 15264254 10.1002/jcc.20084

[26] Doerr S , Harvey MJ , Noé F , De Fabritiis G . HTMD: High‐throughput molecular dynamics for molecular discovery. J Chem Theory Comput. 2016;12(4):1845–1852. 26949976 10.1021/acs.jctc.6b00049

[27] Søndergaard CR , Olsson MHM , Rostkowski M , Jensen JH . Improved treatment of ligands and coupling effects in empirical calculation and rationalization of pKa values. J Chem Theory Comput. 2011;7(7):2284–2295. 26606496 10.1021/ct200133y

[28] Eastman P , Swails J , Chodera JD , et al. OpenMM 7: Rapid development of high performance algorithms for molecular dynamics. PLoS Comput Biol. 2017;13(7):e1005659. 28746339 10.1371/journal.pcbi.1005659 PMC5549999

[29] O'Boyle NM , Banck M , James CA , Morley C , Vandermeersch T , Hutchison GR . Open babel: An open chemical toolbox. J Chem. 2011;3:33. 10.1186/1758-2946-3-33 PMC3198950 21982300

[30] Wang J , Wang W , Kollman PA , Case DA . Automatic atom type and bond type perception in molecular mechanical calculations. J Mol Graph Model. 2006;25(2):247–260. 16458552 10.1016/j.jmgm.2005.12.005

[31] Yesselman JD , Price DJ , Knight JL , Brooks CL . MATCH: An atom‐typing toolset for molecular mechanics force fields. J Comput Chem. 2012;33(2):189–202. 22042689 10.1002/jcc.21963 PMC3228871

[32] Rackers JA , Wang Z , Lu C , et al. Tinker 8: Software tools for molecular design. J Chem Theory Comput. 2018;14(10):5273–5289. 30176213 10.1021/acs.jctc.8b00529 PMC6335969

[33] Ffev . HTMD FFEvaluate–Easy MM force‐field evaluation—HTMD 1.24.8 documentation. [cited 2021 Jul 2]. Available from: https://software.acellera.com/htmd/tutorials/FFEvaluate.html

[34] Hildebrandt A , Dehof AK , Rurainski A , et al. BALL––Biochemical Algorithms Library 1.3. BMC Bioinformatics. 2010;11(1):531. 20973958 10.1186/1471-2105-11-531 PMC2984589

[35] Parmed . ParmEd/ParmEd: Parameter/topology editor and molecular simulator. [cited 2021 Jul 1]. Available from: https://github.com/ParmEd/ParmEd

[36] Kohlbacher O . CADDSuite––A workflow‐enabled suite of open‐source tools for drug discovery. J Chem. 2012;4(S1):1.

[37] Liu Z , Li Y , Han L , et al. PDB‐wide collection of binding data: Current status of the PDBbind database. Bioinformatics. 2015;31(3):405–412. 25301850 10.1093/bioinformatics/btu626

[38] Liu Z , Su M , Han L , et al. Forging the basis for developing protein–ligand interaction scoring functions. Acc Chem Res. 2017;50(2):302–309. 28182403 10.1021/acs.accounts.6b00491

[39] Murkin AS , Birck MR , Rinaldo‐Matthis A , et al. Neighboring group participation in the transition state of human purine nucleoside phosphorylase. Biochemistry. 2007;46(17):5038–5049. 17407325 10.1021/bi700147b PMC2526054

