# Supplementary material for: PocketOptimizer 2.0: A modular framework for computer‐aided ligand‐binding design
Source: Protein Sci. 2023 Jan 1;32(1):e4516. doi: 10.1002/pro.4516 (PMC9793973; doi:10.1002/pro.4516)
Supplement: Supplementary file 1 — Appendix S1: Supporting Information [file PRO-32-e4516-s001.pdf]

Supplementary Information for

PocketOptimizer 2.0: A modular framework for computer-aided ligand-binding design

by Jakob Noske, Josef Paul Kynast, Dominik Lemm, Steffen Schmidt, and Birte Höcker

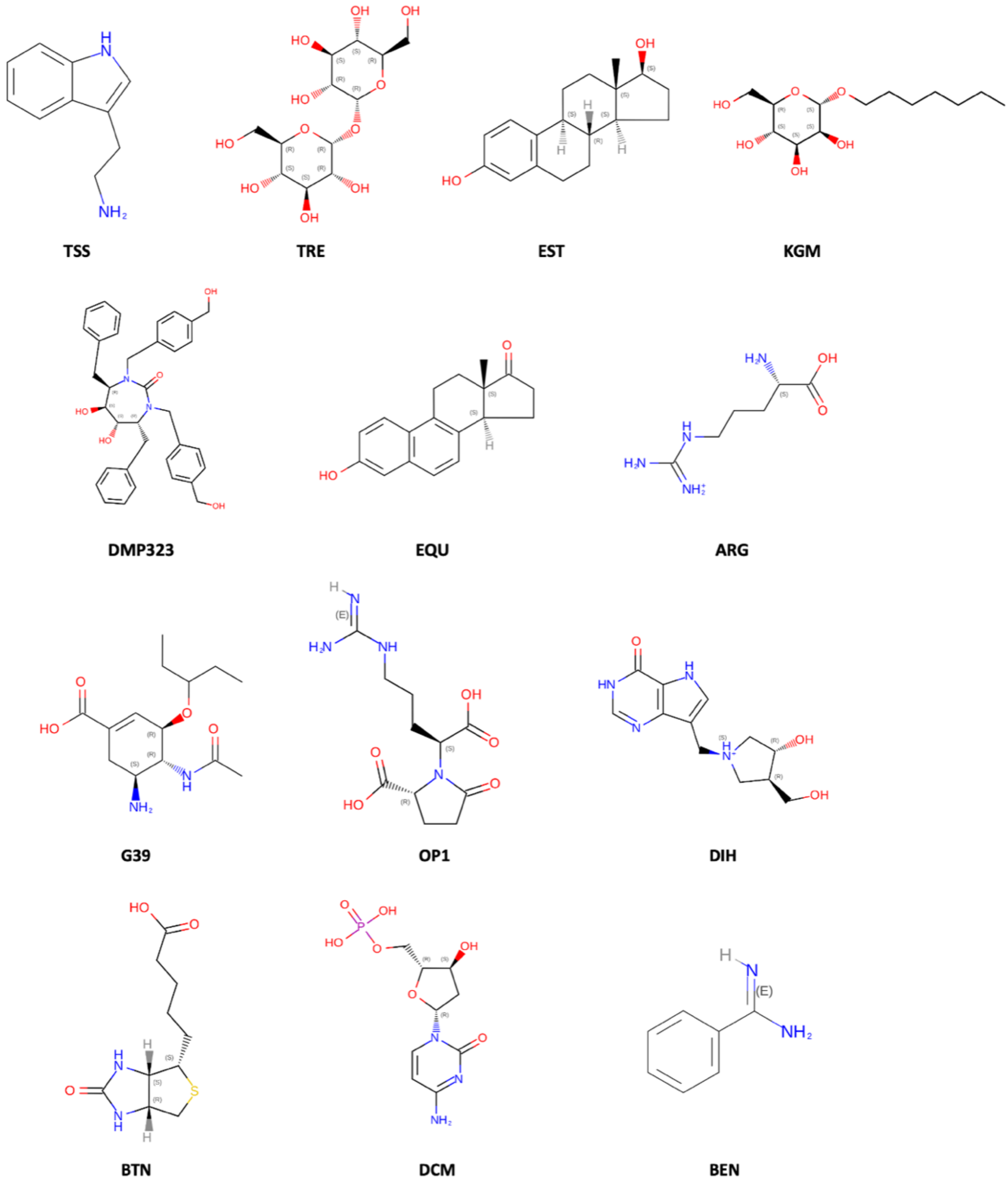

Figure S1: Skeletal representation of ligands included in the benchmark set. The ligand identifiers are shown below, structures were taken from: https://www.rcsb.org.

Table S1: Benchmark used to evaluate PocketOptimizer 2.0 in comparison. It is a subset of the one used to test PocketOptimizer 1.0 and includes all pairs of mutational variants with an affinity change of at least 50-fold. Protein-ligand complexes are sorted by protein names and ligands are listed by their ligand identifiers. Wildtype structures are indicated and all mutations listed. In case of HIV-1 protease, where the binding pocket is formed by two chains, all indicated mutations are present in both chains. The experimentally determined binding affinities for each complex are listed along with the PDB identifier of the experimentally solved structures, if available.

Protein	Ligand	Mutation(s)	Affinity [nM]	PDB
**D7r4 Amine Binding Protein[1]**	TSS	WT	Inf	-
		L111D	53	2pql
**ABC Transporter Alpha-Glycoside-Binding Protein[2]**	TRE	WT	7460	6j9w
		W287A	58	6jb0
**Estrogen Receptor a[3,4]**	EST	WT	0.29	1gwr
		E353A	60	-
**FimH Fimbrial Adhesin[5]**	KGM	WT	1.1	4xo8
		Y137A	206.4	5fs5
**HIV-1 Protease[6]**	DMP323	WT	0.8	-
		V82F	0.4	1met
		I84V	20	1mes
		V82F, I84V	800	1meu
**Ketosteroid Isomerase[7]**	EQU	WT	45750	1oh0
		D40N	810	1ogx
**Lysine-, Arginine-, Ornithine-Binding Periplasmic Protein[8]**	ARG	WT	1.0	6mle
		D11A	70	6mku
		Y14A	800	6mlo
		D30A	5000	6ml9
		R77A	9000	6mlg
		D161A	42000	6mla
**Neuroamidase N1[9]**	G39	WT	0.32	2hu4
		H274Y	84.8	3cl0
		N294S	25.9	3cl2
**Nopaline-Binding Periplasmic Protein[10]**	OP1	WT	0.5	4pow
		M117N	39.9	4pp0

**Purine Nucleoside Phosphorylase[11]**	DIH	WT	0.01	1rsz
		H257D	0.9	2a0y
		H257F	0.95	2a0x
**Streptavidin[12]**	BTN	WT	0.0001	1swe
		N23A	0.028	1n43
		N23E	0.0069	-
		S27A	0.011	1n9m
**Thymidylate Synthase[13]**	DCM	WT	160000	1nje
		N229C	490	1nja
		N229D	2800	1njc
**Anionic Trypsin 2[14]**	BEN	WT	12000	1ane
		D189G, G226D	15000000	1bra

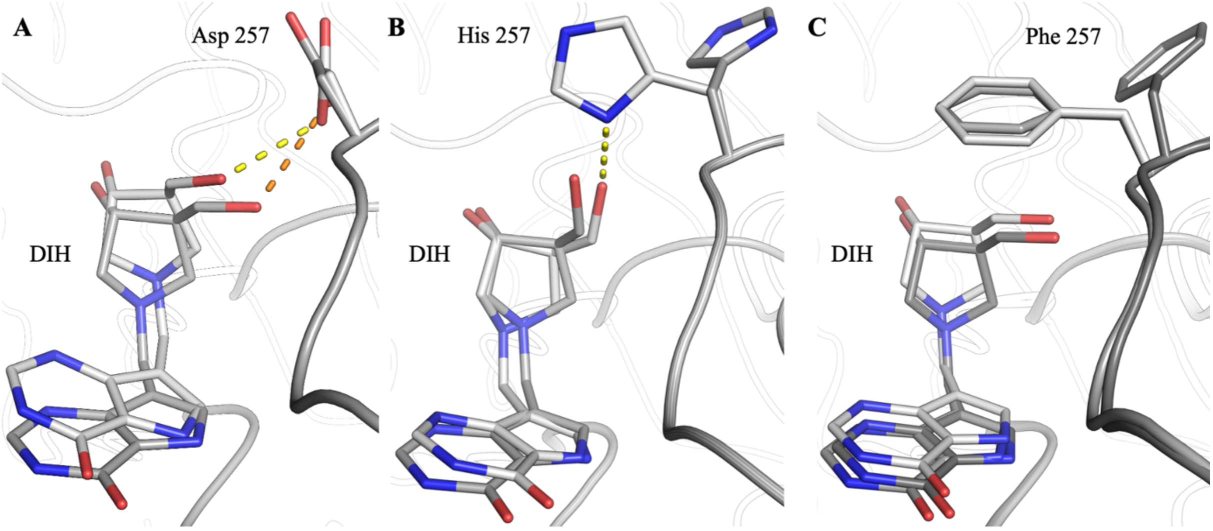

Figure S2: Binding pocket of purine nucleoside phosphorylase with the ligand DIH. Designs based on the original rotamer sampling method and library are shown in light gray, while designs based on the newly implemented rotamer sampling method and library are shown in white. In all designs and crystal structures the conformation of the side chain at mutation position 257 is highlighted. A): Aspartate mutations calculated based on the wildtype crystal structure: 1rsz, hydrogen bonds between aspartate and the ligand are depicted in yellow and orange. B) Histidine mutations calculated based on the mutant crystal structure: 2a0x, hydrogen bonds between histidine and the ligand are depicted in yellow. C) Phenylalanine mutations based on the wildtype crystal structure: 1rsz, the crystal structure of the mutated protein (2a0x) is depicted in dark gray.

References

1. Mans BJ, Calvo E, Ribeiro JMC, Andersen JF. The crystal structure of D7r4, a salivary biogenic amine-binding protein from the malaria mosquito Anopheles gambiae. The Journal of biological chemistry. 2007;282(50):36626–36633.

2. Chandravanshi M, Gogoi P, Kanaujia SP. Structural and thermodynamic correlation illuminates the selective transport mechanism of disaccharide α-glycosides through ABC transporter. The FEBS journal. 2020;287(8):1576–1597.

3. Chen Z, Katzenellenbogen BS, Katzenellenbogen JA, Zhao H. Directed evolution of human estrogen receptor variants with significantly enhanced androgen specificity and affinity. The Journal of biological chemistry. 2004;279(32):33855–33864.

4. Shi Y, Koh JT. Selective regulation of gene expression by an orthogonal estrogen receptor-ligand pair created by polar-group exchange. Chemistry & biology. 2001;8(5):501–510.

5. Rabbani S, Krammer EM, Roos G, Zalewski A, Preston R, Eid S, Zihlmann P, Prévost M, Lensink MF, Thompson A, et al. Mutation of Tyr137 of the universal Escherichia coli fimbrial adhesin FimH relaxes the tyrosine gate prior to mannose binding. IUCrJ. 2017;4(Pt 1):7–23.

6. Ala PJ, Huston EE, Klabe RM, McCabe DD, Duke JL, Rizzo CJ, Korant BD, DeLoskey RJ, Lam PYS, Nicholas Hodge C, et al. Molecular basis of HIV-1 protease drug resistance: structural analysis of mutant proteases complexed with cyclic urea inhibitors. Biochemistry. 1997;36(7):1573–1580.

7. Ha NC, Kim MS, Lee W, Choi KY, Oh BH. Detection of Large pKa Perturbations of an Inhibitor and a Catalytic Group at an Enzyme Active Site, a Mechanistic Basis for Catalytic Power of Many Enzymes. Journal of Biological Chemistry. 2000;275(52):41100–41106.

8. Vergara R, Romero-Romero S, Velázquez-López I, Espinoza-Pérez G, Rodríguez-Hernández A, Pulido NO, Sosa-Peinado A, Rodríguez-Romero A, Fernández-Velasco DA. The interplay of protein-ligand and water-mediated interactions shape affinity and selectivity in the LAO binding protein. The FEBS journal. 2020;287(4):763–782.

9. Collins PJ, Haire LF, Lin YP, Liu J, Russell RJ, Walker PA, Skehel JJ, Martin SR, Hay AJ, Gamblin SJ. Crystal structures of oseltamivir-resistant influenza virus neuraminidase mutants. Nature. 2008;453(7199):1258–1261.

10. Lang J, Vigouroux A, Planamente S, El Sahili A, Blin P, Aumont-Nicaise M, Dessaux Y, Moréra S, Faure D. Agrobacterium uses a unique ligand-binding mode for trapping opines and acquiring a competitive advantage in the niche construction on plant host. PLoS pathogens. 2014;10(10):e1004444.

11. Murkin AS, Birck MR, Rinaldo-Matthis A, Shi W, Taylor EA, Almo SC, Schramm VL. Neighboring group participation in the transition state of human purine nucleoside phosphorylase. Biochemistry. 2007;46(17):5038–5049.

12. Le Trong I, Freitag S, Klumb LA, Chu V, Stayton PS, Stenkamp RE. Structural studies of hydrogen bonds in the high-affinity streptavidin-biotin complex: mutations of amino acids interacting with the ureido oxygen of biotin. Acta crystallographica. Section D, Biological crystallography. 2003;59(Pt 9):1567–1573.

13. Finer-Moore JS, Liu L, Schafmeister CE, Birdsall DL, Mau T, Santi DV, Stroud RM. Partitioning roles of side chains in affinity, orientation, and catalysis with structures for mutant complexes: asparagine-229 in thymidylate synthase. Biochemistry. 1996;35(16):5125–5136.

14. Perona JJ, Tsu CA, McGrath ME, Craik CS, Fletterick RJ. Relocating a negative charge in the binding pocket of trypsin. Journal of molecular biology. 1993;230(3):934–949.
